# Long-chain saturated fatty acid species are not toxic to human pancreatic β-cells and may offer protection against pro-inflammatory cytokine induced β-cell death

**DOI:** 10.1186/s12986-021-00541-8

**Published:** 2021-01-12

**Authors:** Patricia Thomas, Kaiyven A. Leslie, Hannah J. Welters, Noel G. Morgan

**Affiliations:** 1 Institute of Metabolism and Systems Research, Birmingham Medical School, University of Birmingham, Birmingham, UK; 2 Institute of Biomedical and Clinical Research, College of Medicine and Health, University of Exeter, Exeter, UK

**Keywords:** Long-chain fatty acids, Human pancreatic β-cells, Pro-inflammatory cytokines, Lipotoxicity, Type 2 diabetes

## Abstract

Obesity is a major risk factor for type 2 diabetes (T2D) although the causal links remain unclear. A feature shared by both conditions however is systemic inflammation and raised levels of circulating fatty acids (FFA). It is widely believed that in obese individuals genetically prone to T2D, elevated levels of plasma FFA may contribute towards the death and dysfunction of insulin-producing pancreatic β-cells in a process of (gluco)lipotoxicity. In support of this, in vitro studies have shown consistently that long-chain saturated fatty acids (LC-SFA) are toxic to rodent β-cells during chronic exposure (> 24 h). Conversely, shorter chain SFA and unsaturated species are well tolerated, suggesting that toxicity is dependent on carbon chain length and/or double bond configuration. Despite the wealth of evidence implicating lipotoxicity as a means of β-cell death in rodents, the evidence that a similar process occurs in humans is much less substantial. Therefore, the present study has evaluated the effects of chronic exposure to fatty acids of varying chain length and degree of saturation, on the viability of human β-cells in culture. We have also studied the effects of a combination of fatty acids and pro-inflammatory cytokines. Strikingly, we find that LC-FFA do not readily promote the demise of human β-cells and that they may even offer a measure of protection against the toxic effects of pro-inflammatory cytokines. Therefore, these findings imply that a model in which elevated circulating LC-FFA play a direct role in mediating β-cell dysfunction and death in humans, may be overly simplistic.

## Background

The process of fatty acid (FFA) induced β-cell death (frequently referred to as lipotoxicity) is often mooted as a factor mediating the reduction of functional pancreatic β-cell mass in type 2 diabetes (T2D) [1–3]. This is important because there is increasing evidence that obesity and T2D are linked very closely and it has been established that obesity is among the most important risk factors for T2D in genetically predisposed individuals [4]. Thus, it is conceived that an accumulation of abdominal adipose tissue (AT), coupled with decreased sensitivity of peripheral and hepatic tissue to the actions of insulin, causes systemic inflammation and elevated levels of FFA in the blood [5, 6] which are then potentially harmful to pancreatic β-cells. This hypothesis has received ample support from studies performed in vitro, where it is now indisputable evidence that incubation of rodent pancreatic β-cells with long chain saturated fatty acids leads to a loss of viability [1, 7–10].

The accumulation of abdominal AT associated with the onset of obesity in humans has additional consequences in that it is also associated with a decrease in the production of anti-inflammatory mediators (e.g. adiponectin), and an increase in the secretion of pro-inflammatory signalling molecules (e.g. IL-6 and TNF-α) by adipocytes and AT residing immune cells [5, 6]. Moreover, it has become clear by study of pancreas sections recovered at autopsy from subjects with T2D, that islet macrophage infiltration is increased compared to non-diabetic individuals [11]. Taken together, this evidence implies that obesity may be associated with the establishment of an islet milieu favouring β-cell dysfunction and loss since it is well established that pro-inflammatory cytokines can initiate apoptosis in rodent and human β-cells in vitro [7, 12–14]. Given that an expansion of AT leads to the enlargement of adipocytes and to a corresponding increase in the release of FFAs into the blood [15], and that this is associated with an abnormal rise in plasma levels of long-chain saturated fatty acid species (LC-SFA), including palmitate and stearate [16–18], the islet might be considered to lie at the centre of a “perfect storm” in which β-cell demise is favoured.

This scenario may, however, represent an over-simplification of the situation since it is also well-established that not all LC-FFAs are toxic to β-cells. Indeed, a recent epidemiological study [19] has implied that higher circulating concentrations of LC-SFA having odd numbered carbon chains, such as pentadecanoic (C15:0) and heptadecanoic (C17:0) acid, may exert positive effects on β-cells. Moreover, short and medium chain SFA (≤ C14:0) are relatively inert [7] and most LC-MUFA are well tolerated by β-cells. In fact, these fatty acid species appear to exert a cytoprotective influence, by attenuating the toxic effects of palmitate when rodent β-cells are co-exposed to both agents [2, 7, 8]. Furthermore, there is little firm evidence that the progression of T2D is associated with extensive β-cell loss in human subjects and recent studies have revealed that β-cell function can be rapidly regained in response to early and dramatic weight loss [20] or after bariatric surgery in people with T2D [21]. This implies that, while β-cells may become dysfunctional in T2D, they do not die in large numbers. Thus, although the evidence linking chronic LC-FFA exposure to β-cell loss has validity for rodent cells maintained in tissue culture, it is much less clear that a similar situation obtains in humans. Therefore, in the present study, we have examined the effects of a range of LC-FFA on the viability of a human β-cell line, EndoC-βH1, in vitro. We have also studied the effects of exposure of human β-cells to a combination of LC-FFA and pro-inflammatory cytokines.

## Materials and methods

### Cell culture and treatments

Insulin secreting rat-derived INS-1E cells [22] were cultured in RPMI-1640 containing 11 mmol/l glucose (Lonza, Basel, Switzerland) and supplemented with 100 U/ml penicillin and 100 µg/ml streptomycin, 2 mM L-glutamine, 10% foetal bovine serum (FBS) and 50 mmol/l 2-mercaptoethanol (all from ThermoFisher, Boston, MA, USA). EndoC-βH1 cells (sourced from Univercell-Biosolutions) were cultured as detailed in [23], with DMEM or DMEM/F-12 Ham (50%:50% w/w; Merck, Darmstadt, Germany) media containing 5.5 mM glucose and supplemented with 2% (w/v) bovine serum albumin (BSA), 50 mM β-mercaptoethanol, 10 mM Nicotinamide, 100 U/ml penicillin and 100 µg/ml streptomycin, 2 mM L-glutamine, 5.5 µg/ml transferrin and 2.7 nM sodium selenite (sourced as described in [23]). Passage numbers did not exceed 40 and cells routinely tested negative for mycoplasma contamination. For individual experiments, cells were seeded in 12-well plates at a density of 0.5 × 10^6^ cells/well and incubated for 24 h in complete medium. After 24 h, the extracellular medium was removed and replaced with fresh culture medium devoid of FBS or BSA but containing the appropriate LC-FFA/BSA complexes. Where relevant, cells were incubated with proinflammatory cytokines (TNF- α, IL1-β, IFN-γ, IL-6 (20 ng/ml) all from R&D systems, UK).

### Fatty acid preparation

LC-FFA/BSA complexes were prepared in accordance with [8]. Briefly, LC-FFA (Merck) were dissolved in ethanol and conjugated to fatty acid free BSA (Roche, Basal, Switzerland) by incubation at 37 °C for a time period of 1 h. The final concentration of BSA was constant at 1% (w/v) and ethanol used for cell incubations was maintained at 0.5% (v/v). Control cells were treated with vehicle (BSA plus ethanol) only and described interchangeably as 0 µM or vehicle control.

### Cell death analysis by flow cytometry

Cell death was estimated on a BD Accuri™C6 Plus flow cytometer after propidium iodide (Merck; 100) staining as described previously [24]. Experiments were repeated a minimum of 3 times with 2–4 repeats within each experiment.

### Data analysis

Experimental results are expressed as mean + standard error of the mean (SEM). Statistical analysis was performed using GraphPad Prism version 8.0 (https://www.graphpad.com/scientific-software/prism/). Statistical significance between mean values was calculated by one-way analysis of variance (ANOVA) with post hoc Tukey’s test. The difference between groups and control was regarded as significant when *p* < 0.01.

## Results

### Palmitate is selectively toxic to rodent but not human β-cells

**Fig. 1:**
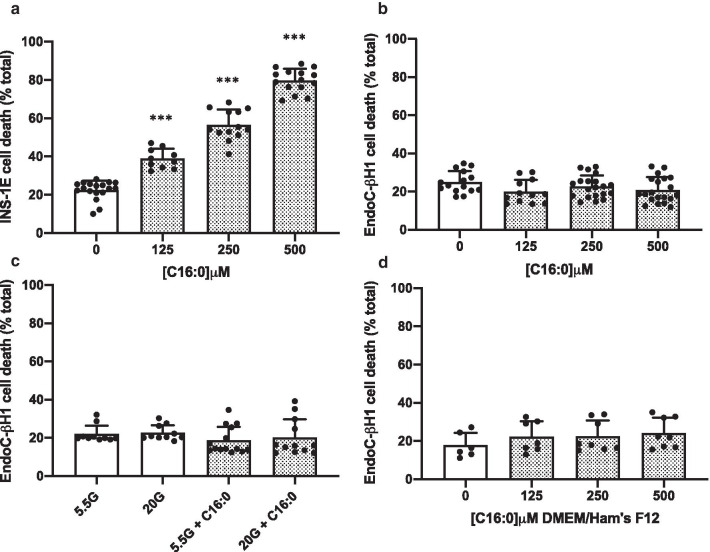
The effect of the LC-SFA, palmitate, on pancreatic β-cell viability. INS-1E (**a**) and EndoC-βH1 (**b**) cells were treated with vehicle [0 µM], 125 µM, 250 µM or 500 µM C16:0 for 24 h or 72 h, respectively. EndoC-βH1 cells were exposed to 5.5 mM or 20 mM glucose (G) with or without 500 µM C16:0 for 72 h (**c**), or incubated for 72 h in DMEM/Ham’s F12 complete culturing medium with increasing concentrations of C16:0 [0–500 µM] (**d**). Cell death was assessed using flow cytometry after staining with propidium iodide. Dots represent individual data points from a minimum of three independent experiments and the histograms represent mean values + SEM. *** p < 0.001 relative to vehicle [0 µM]

Palmitate (C16:0) is the most abundant LC-SFA in the circulation [25], and the effects of chronic exposure (≥ 24 h) of rodent INS-1E and human EndoC-βH1 cells to palmitate were investigated. As expected, C16:0 caused a dose-dependent loss of viability when INS-1E cells were treated with increasing concentrations of C16:0 for 24 h (Fig. 1a). Surprisingly however, over this time course, exposure to C16:0 did not cause any loss in viability of EndoC-βH1 cells. This was also true even when cells were exposed to this fatty acid at concentrations as high as 500 µM, over an extended exposure period of up to 72 h (Fig. 1b). Similarly, C16:0 [500 μM] was well tolerated by EndoC-βH1 cells even in the presence of high glucose [20 mM] (Fig. 1c) which has previously been suggested to exacerbate lipotoxicity in rodent β-cells [3].

In one recent study [26], similar results were obtained but it was proposed that the response varied according to the culture medium in which the cells were grown. Therefore, we substituted the standard growth medium for EndoC-βH1 cells, DMEM, with a second medium, DMEM/Ham’s F12 mixture, since the latter has been proposed to render the cells sensitive to the toxic effects of C16:0 [26]. However, we observed no increase in EndoC-βH1 cell death when cells were treated with C16:0 [500 μM] in DMEM/Ham’s F12 medium (Fig. 1d).

### Effects of other long-chain saturated fatty acids (LC-SFA) on the viability of rodent and human β-cells

**Fig. 2:**
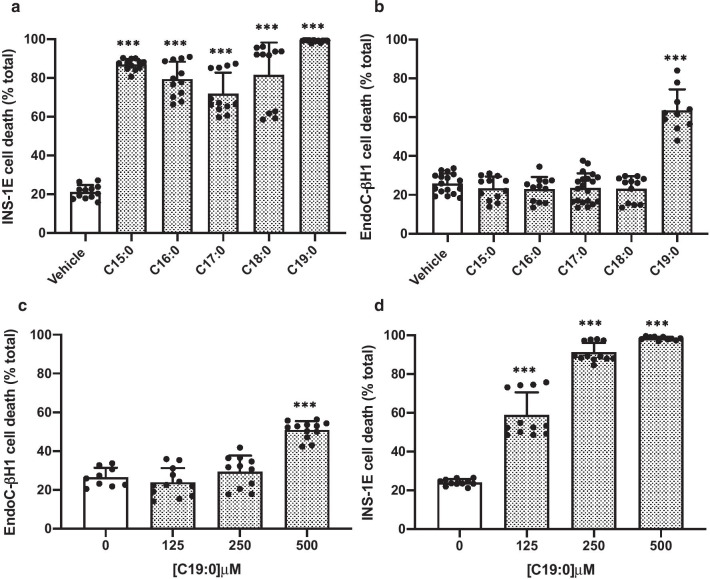
The effect of LC-SFA, increasing in carbon chain length, on β-cell viability. INS-1E (**a**) or EndoC-βH1 (**b**) cells were treated with vehicle [0 µM] or 500 μM of C15:0, C16:0, C17:0, C18:0 or C19:0 for 24 h or 72 h, respectively. EndoC-βH1 (**c**) or INS-1E (**d**) cells were exposed to either vehicle [0 µM], 125 µM, 250 µM or 500 µM C19:0 for 72 h or 24 h, respectively. Cell death was assessed using flow cytometry after staining with propidium iodide. Dots represent individual data points from a minimum of three independent experiments and the histograms represent mean values + SEM. *** p < 0.001 relative to vehicle [0 µM]

Next, we sought to explore if fatty acid chain length was a determinant of the extent of toxicity in rat or human-derived β-cells. Exposure of INS-1E cells to 500 μM of the LC-SFAs, pentadecanoic (C15:0), heptadecanoic (C17:0), stearic (C18:0) and nonadecanoic (C19:0) acid, resulted in a dramatic increase in cell death after 24 h (Fig. 2a). However, toxicity did not correlate directly with chain length, since, when INS-1E cells were incubated with FFAs at the very high concentration of 500 μM, C15:0 caused significantly (*p* = 0.002) greater cell death than was seen with longer chain saturated species. This was also true when a lower concentration [250 μM] was employed (as shown in Additional file 1: Supplementary figure 1).

By contrast with rodent β-cells, human EndoC-βH1 cells were much less sensitive to the detrimental effects of 500 µM of long chain FFAs such as C15:0, C17:0 or C18:0 (Fig. 2b) since their viability was maintained at high levels even after 72 h of treatment. Strikingly, exposure of the EndoC-βH1 cells to C19:0 caused a marked increase (*p* < 0.0001) in death (Fig. 2b) although, even in this case, the lipotoxic effects were less potent than in INS-1E cells (compare Fig. 2c + d). The loss of viability of EndoC-βH1 cells required concentrations of ≥ 500 μM whereas, in INS-1E cells, death was increased significantly at concentrations as low as 125 μM.

### Long-chain monounsaturated fatty acids are well tolerated by both human and rodent β-cells

**Fig. 3:**
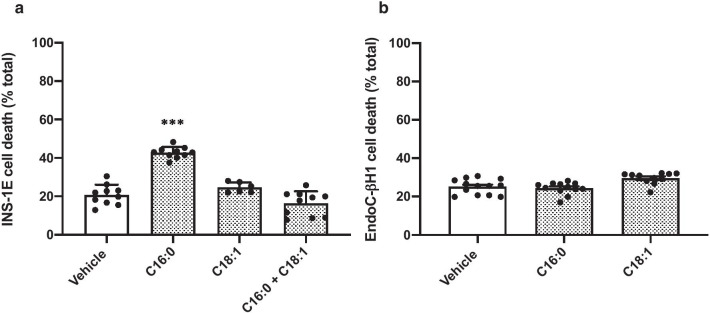
The effect of long-chain monounsaturated fatty acids on β-cell viability. INS-1E (**a**) cells were treated with vehicle [0 µM] or 250 µM C16:0, or C18:1 either alone or in combination [500 µM] for 24 h. EndoC-βH1 cells were exposed to vehicle [0 µM] or 500 µM C16:0 or C18:1 (**b**) for 72 h. Cell death was assessed using flow cytometry after staining with propidium iodide. Dots represent individual data points from a minimum of three independent experiments and the histograms represent mean values + SEM. ***p < 0.001 relative to vehicle [0 µM]

In confirmation of previous studies [7, 24], the long-chain monounsaturated fatty acid (LC-MUFA), oleic acid (C18:1; 250 μM), did not promote any loss of viability in INS-1E cells. Moreover, co-incubation of these cells with C16:0 and C18:1 for 24 h led to an attenuation of the extent of cell death seen with C16:0 alone (Fig. 3a). Similar, to the situation in INS-1E cells, exposure of EndoC-βH1 cells to C18:1 was not associated with any loss of viability (Fig. 3b).

### LC-FFAs attenuate cytokine induced β-cell death in EndoC-βH1 cells

**Fig.4:**
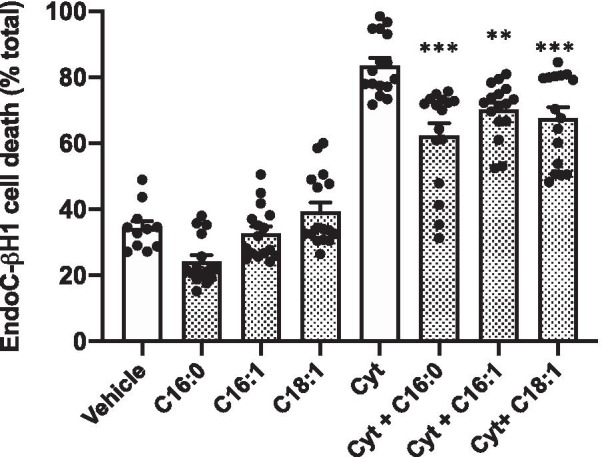
The effect of LC-FFA on β-cell viability when co-incubated with pro-inflammatory cytokines. EndoC-βH1 cells were exposed to vehicle [0 µM] or 250 µM C16:0, C16:1 or C18:1 with and without a cytokine cocktail (cyt; 20 ng TNF-α, IL1-β, IFN-γ, and IL-6) for 48 h. Cell death was assessed using flow cytometry after staining with propidium iodide. Dots represent individual data points from a minimum of three independent experiments and the histograms represent mean values + SEM. **p < 0.01; ***p < 0.001 relative to cytokine [Cyt] vehicle [0 µM LC-FFA] control

We, and others, have previously shown that culture in the presence of a cocktail of pro-inflammatory cytokines induces the loss of viability in rodent β-cells. Moreover, we also discovered that this response was inhibited upon inclusion of the LC-MUFAs, C16:1 or C18:1, in the incubation medium [7]. In the present work, we have confirmed that EndoC-βH1 cells also succumb to pro-inflammatory cytokines (Fig. 4), confirming previous reports by ourselves [7, 12] and others [13]. Surprisingly, however, we found that, when 250 μM of C16:0, C16:1 or C18:1 were also present in the incubation medium, cytokine-induced cell death was attenuated significantly (although, LC-FFAs did not offer complete protection against cytokine induced death under these conditions).

## Discussion

In this study we show that human β-cells are much less sensitive to the “toxic” effects of LC-FFA than rodent β-cells when exposed to FFAs in tissue culture conditions. It is well established that LC-SFA cause the rapid demise of rodent β-cells in culture [1, 7–10] and we have now verified that this applies equally to FFAs having either even or odd-numbered carbon chains. Conversely, we find that LC-SFA were well tolerated by human β-cells, even when present in the culture medium at high concentrations for extended periods of time. We did note, however, that, somewhat paradoxically, C19:0 was capable of initiating cell death when present at high concentrations. The failure of most species of LC-FFA to promote the loss of viability of human β-cells was not altered by the concomitant presence of an elevated glucose concentration (20 mM), implying that glucose does not accentuate lipotoxicity in these cells. It was also unaffected by a change in the culture medium, which differs from the findings of others [26]. Moreover, in our hands, the LC-MUFA, C18:1, was also well tolerated by human β-cells, in contrast to another earlier report [10].

Taken together, these data imply that there are marked differences between the responses of rodent and human β-cells during chronic exposure to LC-FFA and they imply that earlier data obtained with rodent cells may require to be reconsidered before they are simply extrapolated to the human situation. This is especially true when considering circumstances (such as might be found in T2D) when islets are exposed to elevated concentrations of FFA acids in the context of a rise in pro-inflammatory cytokines. Rather than leading to an exacerbation of β-cell loss under these conditions, as might be expected, we found that cell death was attenuated. The mechanisms involved have not been disclosed and warrant further study but the data cast doubt on the likelihood that human β-cell loss is necessarily enhanced when elevations in LC-FFA are coupled with an increase in pro-inflammatory cytokines.

The findings of this study support those of earlier reports [26–28] in which EndoC-βH1 cells were shown to be resistant to C16:0 at concentrations of up to 1 mM during exposure periods of 72 h, both in the absence and presence of high glucose concentrations. However, they are at odds with the work of Plötz et al. who reported that C16:0, C18:0, C19:0 and C18:1 were toxic to EndoC-βH1 cells [10, 29]. Currently, we are uncertain of the reasons for this disparity but it may reflect technical differences since the studies of Plötz et al. mainly used caspase 3 activity measurement as a surrogate for apoptosis although they also compared these with other measures of toxicity. Interestingly, Oshima and colleagues [28] also found that LC-FFAs were well tolerated by EndoC-βH1 cells and proposed that this may be due to elevated expression of the enzyme, stearoyl CoA desaturase (SCD), in a mechanism similar to that which can be achieved in rodent β-cells upon increasing the expression of SCD [30].

To the best of our knowledge, we are the first to characterise the toxicity profile of odd-chain LC-SFA, C15:0 and C17:0 in either human or rodent β-cells. We found no difference in the toxicity profile of odd- and even-chained LC-SFA, and toxicity did not increase with carbon chain length in either rodent or human β-cells. Interestingly, C19:0 did induce EndoC-βH1 cell death, although we also noted that that C19:0 was prone to precipitate in solution, and we cannot exclude that this non-specific physical property may have contributed to the response. This tendency towards precipitation was not observed to the same extent with other LC-SFA.

Our studies have revealed a further unexpected action of LC-FFA in EndoC-βH1 cells since we observed a reduction in the level of cytokine-induced cell death during co-incubation of EndoC-βH1 cells. Although, a similar phenomenon has been observed in clonal rodent β-cells exposed to IL-1β and IFN-γ in the presence of LC-MUFA such as C16:1 or C18:1 [7] an attenuation of cell death has not been attributed previously to the presence of LC-saturated FFA. We found that both monounsaturated and saturated LC-FFAs were cytoprotective under these conditions in EndoC-βH1 cells.

## Conclusion

In summary, the results of our present investigations together with those of others [26–28] suggest that, unlike rodent β-cells, human β-cells do not readily succumb to the toxic effects of LC-FFA. Rather, under certain conditions, LC-FFA may even promote the maintenance of viability in human EndoC-βH1 cells. Future work will be required to determine whether the responses seen in EndoC-βH1 cells recapitulate those of primary human islet cells but, in principle, the present data support the doubts expressed recently by Weir [31] who has cast doubt on the concept that human β-cell loss is favoured under conditions such as those which might be encountered during obesity-induced T2D in humans.

## Supplementary Information

**Additional file 1: Supplementary figure 1**. The effect of exposing rodent β-cells to LC-SFA, increasing in carbon chain length, at a concentration of 250µM for 24h, on viability.

**Additional file 2: Raw datasets**. Raw data (Figures 1–4, sup. fig.1) on which the conclusions of the manuscript rely.

## Data Availability

All data generated or analysed during this study are included in this published article [and its Additional File 2].

## Abbreviations

AT: Adipose tissue
ANOVA: Analysis of variance
BSA: Bovine serum albumin
C15:0: Pentadecanoic acid
C16:0: Palmitic acid
C16:1: Palmitoleic acid
C17:0: Heptadecanoic acid
C18:0: Stearic acid
C18:1: Oleic acid
C19:0: Nonadecanoic acid
DMEM: Dulbecco’s Modified Eagle Medium
FBS: Foetal bovine serum
IFN-γ: Interferon gamma
IL-6: Interleukin-6
IL1-β: Interleukin-1 beta
LC-FFA: Long-chain free fatty acid
LC-MUFA: Long-chain monounsaturated fatty acid
LC-SFA: Long-chain saturated fatty acid
T2D: Type 2 diabetes
TNF-α: Tumor necrosis factor alpha
