# Supplementary material for: Long-chain saturated fatty acid species are not toxic to human pancreatic β-cells and may offer protection against pro-inflammatory cytokine induced β-cell death
Source: Nutr Metab (Lond). 2021 Jan 12;18:9. doi: 10.1186/s12986-021-00541-8 (PMC7802137; doi:10.1186/s12986-021-00541-8)
Supplement: Supplementary file 1 — Additional file 1: Supplementary figure 1. The effect of exposing rodent β-cells to LC-SFA, increasing in carbon chain length, at a concentration of 250µM for 24h, on viability. [file 12986_2021_541_MOESM1_ESM.pdf]

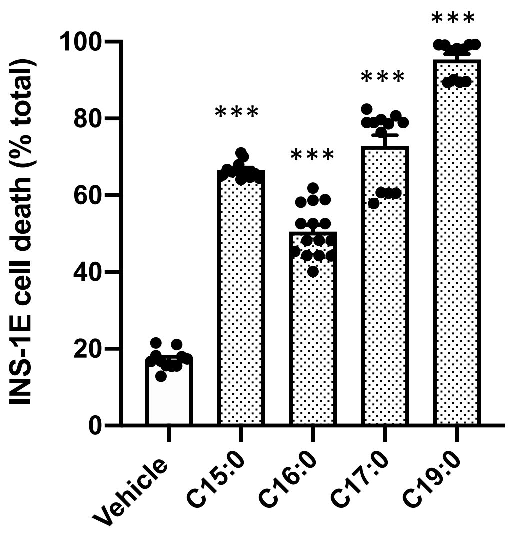

Supplementary Figure 1: The effect of LC-SFA, increasing in carbon chain length, on rodent β-cell viability. INS-1E cells were treated with vehicle [0 µM] or 250µM of C15:0, C16:0, C17:0 or C19:0 for 24h. Cell death was assessed using flow cytometry after staining with propidium iodide. Dots represent individual data points from a minimum of three independent experiments and the histograms represent mean values + SEM. ***p<0.001 relative to vehicle [0 µM].
